# Elevated hair cortisol concentrations in recently fled asylum seekers in comparison to permanently settled immigrants and non-immigrants

**DOI:** 10.1038/tp.2017.14

**Published:** 2017-03-07

**Authors:** R Mewes, H Reich, N Skoluda, F Seele, U M Nater

**Affiliations:** 1 Division of Clinical Psychology and Psychotherapy, Department of Psychology, University of Marburg, Marburg, Germany; 2 Division of Clinical Biopsychology, Department of Psychology, University of Marburg, Marburg, Germany

## Abstract

Recently fled asylum seekers generally live in stressful conditions. Their residency status is mostly insecure and, similar to other immigrants, they experience stress due to acculturation. Moreover, they often suffer from traumatization and posttraumatic stress disorder (PTSD). All of these factors can result in chronic maladaptive biological stress responses in terms of hyper- or hypocortisolism and, ultimately, illness. We believe the current study is the first to compare hair cortisol concentration (HCC) of recently fled asylum seekers with PTSD to those without PTSD, and to compare HCC of asylum seekers to HCC of permanently settled immigrants and non-immigrant individuals. HCC of the previous 2 months was compared between 24 asylum seekers without PTSD, 32 asylum seekers with PTSD, 24 permanently settled healthy Turkish immigrants and 28 non-immigrant healthy Germans as the reference group. Statistical comparisons were controlled for age, sex and body mass index. No significant difference in HCC was found between asylum seekers with and without PTSD. However, the asylum seekers showed a 42% higher HCC than the reference group. In contrast, the permanently settled immigrants exhibited a 23% lower HCC than the reference group. We found relative hypercortisolism in recently fled asylum seekers, but no difference between persons with and without PTSD. These findings add to the very few studies investigating HCC in groups with recent traumatization and unsafe living conditions. Contrary to the findings in asylum seekers, permanently settled immigrants showed relative hypocortisolism. Both hyper- and hypocortisolism may set the stage for the development of stress-related illnesses.

## Introduction

Over the past few years, more than 60 million individuals have been forcibly displaced worldwide,^1^ including persons displaced within a country, refugees and asylum seekers. Most flee in order to escape from situations threatening their own lives or the lives of their family members through armed attacks, torture or sexual violence. Moreover, the severe traumatic events experienced in the home country are often compounded by traumatic events during the highly risky process of fleeing. Even in secure host countries, refugees applying for asylum (asylum seekers) experience insecure living conditions (for example, an uncertain future) and post-migration living difficulties such as language problems, ethnic discrimination, being separated from family members and having limited access to health care services, which cumulatively add to a generally high stress level.^2, 3, 4, 5^ Considering the very stressful living conditions of asylum seekers and the negative impact of chronic stress on mental and physical health,^6, 7^ it is imperative to investigate biological markers of stress and impaired health in asylum seekers.^8^

The hypothalamic–pituitary–adrenal (HPA) axis, with cortisol as its main end product,^9^ is frequently discussed as the underlying biological link between the experience of stress and the manifestation of illness.^10^ However, there is a general lack of studies investigating HPA axis activity in asylum seekers. The very few studies that measured cortisol in the blood, urine or saliva of asylum seekers yielded inconsistent findings. For instance, Bauer *et al.*^11^ and Rohleder *et al.*,^12^ found lower cortisol concentrations in serum^11^ and saliva^12^ in comparison to healthy controls, but not in urine.^12^ In contrast, Sabioncello *et al.*^13^ found higher serum cortisol levels in displaced women in comparison to a non-displaced control group. However, these studies differ in their methodological approaches and the investigated sample characteristics. Moreover, they allow only limited conclusions about longer-lasting HPA axis activity due to their rather short-term assessment of cortisol. Considering the cumulated threatening events and ongoing insecure living conditions of asylum seekers, a long-term measure of cortisol might be more appropriate to reflect the accompanying biological alterations. Hair cortisol concentration (HCC) provides such a long-term measure as it allows the retrospective assessment of cumulative cortisol secretion over a time period up to several months.^14^

The investigation of cortisol in asylum seekers is further complicated by the high prevalence rates of posttraumatic stress disorder (PTSD) in this group.^15, 16, 17^ The few existing studies on HCC in traumatized persons with or without PTSD showed inconsistent results. Steudte *et al.*^18^ found increased HCC in severely traumatized Ugandan individuals with PTSD compared to traumatized individuals without PTSD. These individuals were still in an insecure situation (living in a camp for internally displaced people) and their traumatic experiences had occurred only recently. Similarly, Luo *et al.*^19^ found increased HCC in victims of a recent earthquake in comparison to non-exposed individuals. However, ~6 months after the earthquake, this effect was reversed, with those individuals with PTSD showing lower HCC.^19^ The latter finding is in line with the results of the study by Steudte *et al.*^20^ In a sample of individuals whose traumatic experiences dated back 5 years or more, decreased HCC was found in comparison to a non-traumatized control group. Taken together, the literature suggests that recently traumatized persons and persons living in insecure conditions suffer from relatively high HCC (relative hypercortisolism), whereas persons whose traumatic experiences occurred longer ago or persons with PTSD living in secure conditions display relatively low HCC (relative hypocortisolism). It has been proposed that such a change was a result of the counter-regulatory processes of the HPA axis and might also represent specific pathophysiological processes of neuroendocrine and immune changes following trauma and chronic stress.^21, 22, 23^ However, more studies are needed to shed light on this issue. Moreover, so far, no study has investigated HCC in asylum seekers with and without PTSD.

When investigating the possible impact of traumatic events and diagnosis of PTSD on HCC in asylum seekers, it is important to control for the possible impact of stressful post-migration living difficulties. Therefore, healthy permanently settled immigrants are needed as a comparison group. They share the impact of post-migration living difficulties, such as language problems, ethnic discrimination or stress due to acculturation,^24^ but do not suffer from insecure living conditions and recent traumatization. Moreover, healthy non-immigrant persons are needed as a second comparison group, providing an anchor level for HCC.

To the best of our knowledge, the current study is the first study to investigate HCC in asylum seekers and immigrants. It thus had two aims: first, to compare HCC, as a long-term marker for the endocrine stress response, of recently fled asylum seekers with PTSD to those without PTSD, and second, to compare HCC of asylum seekers to HCC of permanently settled immigrants and non-immigrant individuals. We hypothesized that recently fled asylum seekers with PTSD would show higher HCC than asylum seekers without PTSD, and that asylum seekers in general would show higher HCC than the two comparison groups.

## Materials and methods

The study was approved by the local ethics committee, and all subjects provided written informed consent before participating in the study. The study was conducted in accordance with the Declaration of Helsinki.

### Participants and procedure

HCC accumulated over the previous 2 months was investigated in 24 asylum seekers without PTSD and 32 asylum seekers with PTSD in order to address the first study aim. Asylum seekers had been living in Germany for an average of 7 months (s.d.=5, range: 1–24 months). We included both men and women in order to investigate samples that are as representative as possible for the whole group of asylum seekers. Moreover, studies in asylum seekers show that women are often more severely affected by traumatic experiences than men, and are thus an important group to investigate in this context.^15^

For the second study aim, two male samples were recruited, that is, 24 healthy permanently settled Turkish immigrants and 28 healthy non-immigrant Germans as the reference group (Table 1). These two groups were compared to male asylum seekers only, because neurobiological responses to stress differ between men and women^25^ and the menstrual cycle-related changes in women's hormonal state further influence cortisol secretion.^26^ Therefore, we decided to control for those influences by only investigating men in the comparison groups.

Asylum seekers living in Hesse, Germany, were approached in their accommodation, and hair samples were collected (see sample collection section below). In addition, traumatic events and posttraumatic symptoms were assessed via self-report using the Posttraumatic Diagnostic Scale (PDS^27^). The trauma scale of the PDS was extended by adding items from the Harvard Trauma Questionnaire asking about traumatic events often experienced by refugees.^28^ All questions were provided in Farsi, Arabic, Kurdish or English according to the participants' choice, and trained translators for these languages were present in case of questions about to the linguistic meaning of the items. In addition, a member of the research team was always available in case of further questions regarding the content of a question or other questions associated with the study. Thoroughly trained clinical psychologists, with the help of specifically trained translators, assessed the diagnosis of PTSD. To this end, the respective section of the Structured Clinical Interview for DSM-IV (SCID-I^29^) was used and the translators translated everything said by the interviewer and the interviewee on a word-by-word basis during the interview. The translators were native speakers of the respective languages with very good verbal and written knowledge of the German language. They were also consulted in case of uncertainties about the classification of reported symptoms with regard to the cultural backgrounds and cultural norms.

All asylum seekers had experienced traumatic events such as torture, violence or sexual abuse. Twenty-five asylum seekers fulfilled the diagnostic criteria for PTSD (asylum seekers with PTSD) and 20 did not fulfill the criteria (asylum seekers without PTSD). A subset of 11 asylum seekers were not eligible for the SCID (for example, due to time constraints or feeling unwell). These asylum seekers were assigned to the ‘with PTSD' or ‘without PTSD' groups based on their summary score on the PDS symptom scale (all asylum seekers had experienced at least one traumatic event, thereby fulfilling the gate criterion for PTSD). Further analyses showed no significant differences with regard to age, body mass index (BMI), HCC, frequency of hair washes per week, and percentages of persons with curls or waves (all *t*-test or *χ*^2^-tests *P*>0.05) in the group of asylum seekers diagnosed with PTSD based on the SCID (*n*=25) in comparison to the persons categorized based on the PDS (*n*=7). The percentage of women was descriptively higher in the PDS group (86%) than in the SCID group (48% *χ*^2^_1_=3.16; *P*=0.075). Besides, we found a significantly higher PDS mean value in the PDS group (*M*=41.7 (s.d.=6.9)) than in the SCID group (*M*=32.5 (s.d.=14.7); *t*(22.5)=−2.22; *P*=0.036), and more persons with hair coloration in the PDS group (86%) than in the SCID group (20% *χ*^2^_2_=10.58; *P*=0.005). The asylum seekers without PTSD (*N*=24; *n*=20 with SCID and *n*=4 with PDS) did not differ with regard to age, gender, BMI, HCC, PDS mean values or hair-related variables (all *P*>0.05).

Permanently settled Turkish immigrants and non-immigrant Germans (men only) were approached through advertising in public places and in a local online journal, as well as through mailing lists of the local university, social networks (all documents were available in Turkish and German) or through personal contacts. Interested individuals underwent a thorough screening regarding the eligibility criteria. The Turkish immigrants themselves or at least one of their parents had to have been born in Turkey. The participants of the comparison groups were physically and mentally healthy.

### Sample collection

Several thin hair strands were cut as closely as possible to the scalp from a posterior vertex position of the head. The first 2-cm segment closest to the scalp was used for hair cortisol analyses, which is thought to reflect the cumulative cortisol secretion of the past 2 months.^30^ As the majority of our participants were males who tend to have shorter hair, we chose to investigate cortisol in 2 cm hair strands instead of the more common (but also stricter) criterion of a minimum hair length of 3 cm. Thereby, we were able to include as many participants of the groups of interest as possible and also minimize a possible selection bias.

Hair-washing and cortisol extraction procedures were based on the laboratory protocol by Stalder *et al.*,^14^ with minor modifications. In brief, hair samples were washed twice by shaking them for 3 min using 3 ml isopropanol. For cortisol extraction, 10±0.5 mg of each sample were finely cut and incubated in 1.8 ml methanol for 18 h at room temperature. Then, 1.6 ml of the supernatant was evaporated at 50 °C until samples were completely dried. Finally, the samples were resuspended with 150 μl HPLC gradient grade water (Fisher Scientific, Schwerte, Germany) and vortexed for 20 s. All samples were stored at −20 °C until assayed. For cortisol determination, 50 μl was used for analysis with a commercially available luminescence immunoassay (IBL, Hamburg, Germany). Inter- and intra-assay coefficients of variation were below 10% for all assays. The analyses were carried out at our local laboratory (Clinical Biopsychology, University of Marburg).

### Statistical analyses

For the first study aim, asylum seekers with PTSD were compared to those without PTSD using analysis of covariance (ANCOVA) controlling for sex, age and BMI. For the second study aim, group comparisons were conducted with ANCOVA controlling for age and BMI (only men were included in this analysis). For variables with significant results regarding HCC in the ANCOVA, Bonferroni-corrected pairwise comparisons were conducted *post hoc*. The Shapiro–Wilk test as well as inspections of the Q–Q plots confirmed normal distribution of residuals for our dependent variable. The Levene test showed variance homogeneity for the comparison of asylum seekers with PTSD to those without PTSD. Variance homogeneity was not fulfilled for the comparison of asylum seekers, immigrants and non-immigrants Germans. However, ANCOVAs are robust against such violations when the sample sizes are relatively equal, as is the case for the present study. Therefore, we used an ANCOVA for this analysis.

## Results

**Figure 1:**
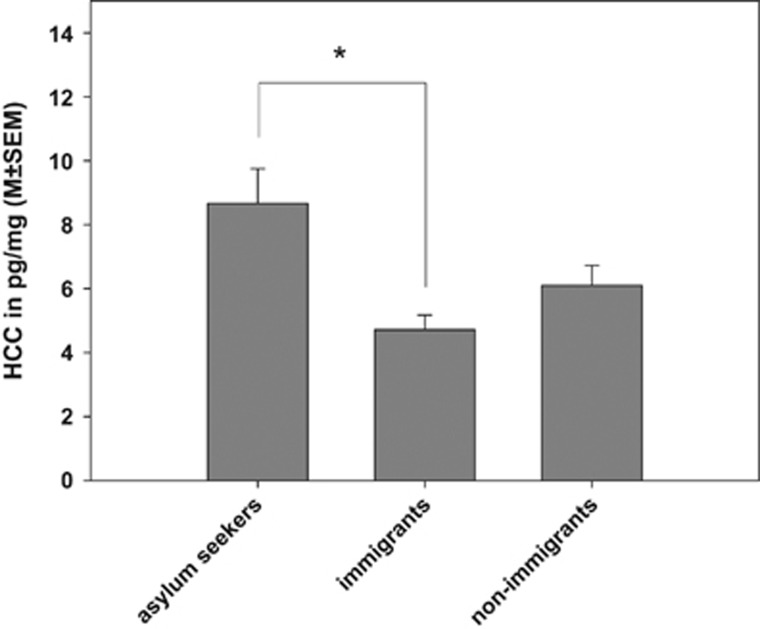
Mean (±s.e.m.) cortisol concentrations in the first 2-cm hair segment of asylum seekers, immigrants and non-immigrant Germans. *P<0.05. HCC, hair cortisol concentrations.

The comparison of asylum seekers with and without PTSD yielded no significant difference between the groups (F_1,51_=1.07; *P*=0.38). Therefore, male asylum seekers with and without PTSD were combined for the comparison with the two comparison groups. The simultaneous group comparison showed significant differences between the groups (F_2,75_=3.56; *P*=0.03). BMI was a significant control variable (*P*=0.03). HCC was highest in asylum seekers (8.67 pg/mg; 42% increase compared to the non-immigrant reference group; see Figure 1). In contrast, the permanently settled immigrants exhibited 23% lower HCC compared to the non-immigrant reference group. However, in the pairwise comparisons, only the difference between asylum seekers and permanently settled immigrants was significant (*P*=0.03).

## Discussion

This is the first study to investigate HCC in asylum seekers with and without PTSD, and to compare it with that of permanently settled immigrants. Consistent with our hypothesis, we found relative hypercortisolism in recently fled asylum seekers. However, we found no difference between those with and without PTSD. Furthermore, we found relative hypocortisolism in permanently settled immigrants. Through the complex influences of cortisol secretion on different endpoints in physiological systems, such as the immune system or the cardiovascular system, both hyper- or hypocortisolism may set the stage for the development of stress-related illnesses.

Our findings of relative hypercortisolism in recently fled asylum seekers add to and are in line with the very few studies investigating HCC in groups with recent or ongoing traumatization and insecure living conditions.^18^ However, we found no difference in HCC between asylum seekers with and without PTSD. This might be explained by the insecure living conditions of the asylum seekers that participated in our study. In line with the findings of Heeren and colleagues,^31, 32^ we assume that the legal status as a recently arrived asylum seeker in a fragile legal situation regarding the residency permit was associated with higher psychopathology in both groups with and without PTSD. This finding is in contrast to the results of Steudte *et al.*^18^ in traumatized Ugandan individuals living in a camp for internally displaced persons. This discrepancy might be explained by differences in the investigated groups and their living conditions (internally displaced persons versus recently fled asylum seekers in a fragile legal situation; no posttraumatic symptoms in the control group of Steudte *et al.*^18^ and the older sample investigated in the present study^33^). Moreover, we found relative hypocortisolism in Turkish immigrants. It is conceivable that Turkish immigrants experience chronic stress due to acculturation and ethnic discrimination.^34^ While many asylum seekers experienced chronic stress in their country of origin for many years as well, all asylum seekers experienced at least one traumatic event prior to or during their flight, irrespective of whether they fulfilled the other diagnostic criteria of PTSD or not. Therefore, the differences between asylum seekers and Turkish immigrants can be explained by traumatic experiences/traumatic stress that can alter cortisol expression in the long term together with the extremely stressful insecure living conditions discussed above. It has been proposed that chronic stress is marked by initial hypercortisolism, which later turns into hypocortisolism as a result of the counter-regulatory processes of the HPA axis.^21, 22^ For instance, hypocortisolism has been reported in healthy individuals who suffered from childhood trauma.^35, 36^ However, altered HPA axis regulation most likely develops as an interaction of both a genetic disposition and stressful or traumatic events. It would be of great interest to determine the physiological underpinnings of our observations; it is conceivable, for example, that altered cortisol regulation may be explained by polymorphisms in FKBP5, which serves as a functional regulator of the glucocorticoid receptor (which in turn is instrumental in regulating negative feedback of the HPA axis).^37^ In the current study, we were not able to determine genetic disposition, so future studies should complement peripheral measurements with molecular assessments. Despite this limitation, the presented results support the hypothesis that strong and more recent stress as well as living in insecure conditions can cause relative hypercortisolism, whereas chronic ongoing stress may ultimately result in hypocortisolism.

The determination of hair cortisol is a novel method that presents a biological correlate of the accumulated cortisol production during the last months. Possibly, HCC is sensitive to different stages of stress-related illnesses, with hyper- and hypocortisolism representing specific pathophysiological processes of neuroendocrine and immune changes following trauma and chronic stress.^23^ Therefore, the determination of HCC might be helpful in selecting appropriate treatment strategies. Against this background, recently fled asylum seekers showing hypercortisolism would benefit from safe living conditions in the recipient countries. In this regard, it seems to be imperative to reduce acute stress due to insecure living conditions and post-migration living difficulties.

The current study has limitations. First, the sample sizes were relatively small, although they are comparable to or even larger than those of other studies on HCC in traumatized individuals. Asylum seekers are extremely difficult to recruit due to their insecure living conditions, their often rural and shielded accommodation, language difficulties and their—understandable—distrust of any formal organizations that may threaten their asylum process. Second, the study is also limited by the differences between the investigated groups regarding age and BMI, although we statistically controlled for these differences. In addition, future studies should consider additional factors that possibly affect HCC. For instance, physical activity was found to be positively associated with HCC (for example, Ullmann *et al.*^8, 38^). Third, the necessary but strict selection procedure for the comparison groups might have influenced the results. For instance, because of the exclusion of physically ill persons, stress or impairments associated with a physical illness could not be accounted for in the comparisons.

The presented findings illustrate the biological impact of traumatic events, the recent flight, and the stressful living conditions on asylum seekers and may inform appropriate treatment strategies. Future studies may take a closer look at the longitudinal course of the biological alterations.

## Figures and Tables

**Table 1:** Sociodemographic characteristics and hair-related data

	*Asylum seekers with PTSD (*n=*32)*	*Asylum seekers without PTSD (*n=*24)*	*Turkish immigrants (*n=*24)*	*Non-immigrant Germans (*n=*28)*	*Group differences F*_*df*_*/*χ^*2*^_*df*_*;* P
Age (years): mean±s.d. (range)	32.8±6.9 (20–48)	32.0±7.6 (20–49)	24.3±2.7 (20–30)	25.9±3.8 (20–36)	F_3,104_=15.2; *P*⩽0.001

*Sex: men*					
Sex: men n (%)^a^	14 (44%)	14 (58%)	24 (100%)	28 (100%)	*χ*^2^_1_=1.2^a^; *P*=0.28
BMI (kg/m^2^): mean±s.d.	26.6±5.1	24.0±3.2	26.2±3.5	22.8±2.2	F_3,104_=6.4; *P*⩽0.001
Born in Germany	0%	0%	73.3%	100%	Not appropriate
German citizenship	0%	0%	70.0%	100%	Not appropriate
*Born in*^a^					*χ*^2^_5_=6.3^a^; *P*=0.28
Iran	50%	62.5%	—	—	
Afghanistan	9%	21%	—	—	
Syria	25%	4%	—	—	
Eritrea, Somalia, Kosovo	16%	12.5%	—	—	

*Hair-related variables*					
Washes per week	4.8 (2.6)	4.4 (2.0)	5.4 (1.5)	5.6 (1.9)	F_3,103_=2.8; *P*=0.045
Curls/waves	55%	71%	46%	46%	*χ*^2^_6_=5.3; *P*=0.51
Permanent waves	3%	0%	0%	0%	*χ*^2^_3_=2.5; *P*=0.48
Coloration	47%	46%	0%	0%	*χ*^2^_6_=38.7; *P*⩽0.001
					
*Two-month hair cortisol (pg /mg):*					
Mean±s.d. (range)	8.21±4.62 (1.55–21.89)	7.43±4.93 (2.29–25.06)	4.72±2.23 (1.67–11.29)	6.10±3.30 (1.50–15.69)	F_3,104_=4.0; *P*=0.009
Only men: mean±s.d. (range)	8.58±5.90 (1.84–21.89)	8.75±5.77 (2.65–25.06)	See above	See above	F_2,75_=3.56; *P*=0.03

Abbreviations: BMI, body mass index; PTSD, posttraumatic stress disorder.

a Note: group comparison (*χ*^2^-test) between asylum seekers with PTSD versus asylum seekers without PTSD.

## References

[bib1] United Nations High Commissioner for Refugees. Mid-Year Trends 2015. Genf 2015. Available at: http://www.unhcr.org/statistics/unhcrstats/56701b969/mid-year-trends-june-2015.html.

[bib2] Laban CJ, Gernaat HB, Komproe IH, Schreuders BA, De Jong JT. Impact of a long asylum procedure on the prevalence of psychiatric disorders in Iraqi asylum seekers in The Netherlands. J Nerv Ment Dis 2004; 192: 843–851. 15583506 10.1097/01.nmd.0000146739.26187.15

[bib3] Porter M, Haslam N. Predisplacement and postdisplacement factors associated with mental health of refugees and internally displaced persons: a meta-analysis. JAMA 2005; 294: 602–612. 16077055 10.1001/jama.294.5.602

[bib4] Silove D, Sinnerbrink I, Field A, Manicavasagar V, Steel Z. Anxiety, depression and PTSD in asylum-seekers: assocations with pre-migration trauma and post-migration stressors. Br J Psychiatry 1997; 170: 351–357. 9246254 10.1192/bjp.170.4.351

[bib5] Steel Z, Silove D, Brooks R, Momartin S, Alzuhairi B, Susljik I. Impact of immigration detention and temporary protection on the mental health of refugees. Br J Psychiatry 2006; 188: 58–64. 16388071 10.1192/bjp.bp.104.007864

[bib6] Kemeny ME. Psychobiological responses to social threat: evolution of a psychological model in psychoneuroimmunology. Brain Behav Immun 2009; 23: 1–9. 18809488 10.1016/j.bbi.2008.08.008

[bib7] McEwen BS. Protective and damaging effects of stress mediators. N Engl J Med 1998; 338: 171–179. 9428819 10.1056/NEJM199801153380307

[bib8] Ullmann E, Barthel A, Taché S, Bornstein A, Licinio J, Bornstein SR. Emotional and psychological trauma in refugees arriving in Germany in 2015. Mol Psychiatry 2015; 20: 1483–1484. 26527128 10.1038/mp.2015.164

[bib9] Charmandari E, Tsigos C, Chrousos G. Endocrinology of the stress response. Annu Rev Physiol 2005; 67: 259–284. 15709959 10.1146/annurev.physiol.67.040403.120816

[bib10] Chrousos GP. Stress and disorders of the stress system. Nat Rev Endocrinol 2009; 5: 374–381. 19488073 10.1038/nrendo.2009.106

[bib11] Bauer M, Priebe S, Gräf KJ, Kürten I, Baumgartner A. Psychological and endocrine abnormalities in refugees from East Germany: Part II. Serum levels of cortisol, prolactin, luteinizing hormone, follicle stimulating hormone, and testosterone. Psychiatry Res 1994; 51: 75–85. 8197272 10.1016/0165-1781(94)90048-5

[bib12] Rohleder N, Joksimovic L, Wolf JM, Kirschbaum C. Hypocortisolism and increased glucocorticoid sensitivity of pro-Inflammatory cytokine production in Bosnian war refugees with posttraumatic stress disorder. Biol Psychiatry 2004; 55: 745–751. 15039004 10.1016/j.biopsych.2003.11.018

[bib13] Sabioncello A, Kocijan-Hercigonja D, Rabatić S, Tomasić J, Jeren T, Matijević L et al. Immune, endocrine, and psychological responses in civilians displaced by war. Psychosom Med 2000; 62: 502–508. 10949095 10.1097/00006842-200007000-00008

[bib14] Stalder T, Steudte S, Alexander N, Miller R, Gao W, Dettenborn L et al. Cortisol in hair, body mass index and stress-related measures. Biol Psychol 2012; 90: 218–223. 22476032 10.1016/j.biopsycho.2012.03.010

[bib15] Johnson H, Thompson A. The development and maintenance of post-traumatic stress disorder (PTSD) in civilian adult survivors of war trauma and torture: a review. Clin Psychol Rev 2008; 28: 36–47. 17383783 10.1016/j.cpr.2007.01.017

[bib16] Fazel M, Wheeler J, Danesh J. Prevalence of serious mental disorder in 7000 refugees resettled in western countries: a systematic review. Lancet 2005; 365: 1309–1314. 15823380 10.1016/S0140-6736(05)61027-6

[bib17] Steel Z, Chey T, Silove D, Marnane C, Bryant RA, van Ommeren M. Association of torture and other potentially traumatic events with mental health outcomes among populations exposed to mass conflict and displacement: a systematic review and meta-analysis. JAMA 2009; 302: 537–549. 19654388 10.1001/jama.2009.1132

[bib18] Steudte S, Kolassa IT, Stalder T, Pfeiffer A, Kirschbaum C, Elbert T. Increased cortisol concentrations in hair of severely traumatized Ugandan individuals with PTSD. Psychoneuroendocrinology 2011; 36: 1193–1200. 21411229 10.1016/j.psyneuen.2011.02.012

[bib19] Luo H, Hu X, Liu X, Ma X, Guo W, Qiu C et al. Hair cortisol level as a biomarker for altered hypothalamic-pituitary-adrenal activity in female adolescents with posttraumatic stress disorder after the 2008 Wenchuan earthquake. Biol Psychiatry 2012; 72: 65–69. 22305287 10.1016/j.biopsych.2011.12.020

[bib20] Steudte S, Kirschbaum C, Gao W, Alexander N, Schönfeld S, Hoyer J et al. Hair cortisol as a biomarker of traumatization in healthy individuals and posttraumatic stress disorder patients. Biol Psychiatry 2013; 74: 639–646. 23623187 10.1016/j.biopsych.2013.03.011

[bib21] Fries E, Hesse J, Hellhammer J, Hellhammer DH. A new view on hypocortisolism. Psychoneuroendocrinology 2005; 30: 1010–1016. 15950390 10.1016/j.psyneuen.2005.04.006

[bib22] Miller GE, Chen E, Zhou ES. If it goes up, must it come down? Chronic stress and the hypothalamic-pituitary-adrenocortical axis in humans. Psychol Bull 2007; 133: 25–45. 17201569 10.1037/0033-2909.133.1.25

[bib23] Wieck A, Grassi-Oliveira R, Hartmann do Prado C, Teixeira AL, Bauer ME. Neuroimmunoendocrine interactions in post-traumatic stress disorder: focus on long-term implications of childhood maltreatment. Neuroimmunomodulation 2014; 21: 145–151. 24557048 10.1159/000356552

[bib24] Berry JW. Immigration, acculturation, and adaptation. Appl Psychol 1997; 46: 5–68.

[bib25] Taylor SE, Klein LC, Lewis BP, Gruenewald TL, Gurung RA, Updegraff JA. Biobehavioral responses to stress in females: tend-and-befriend, not fight-or-flight. Psychol Rev 2000; 107: 411–429. 10941275 10.1037/0033-295x.107.3.411

[bib26] Strahler J, Nater UM. Social stress: sex-related differences in biological stress responses. In: Advances in Medicine and Biology, vol. 104; Invited chapter. Hauppauge NY: Nova Publisher; in press.

[bib27] Foa E. Posttraumatic Stress Diagnostic Scale Manual. Minneapolis: National Computer Systems Inc.;, 1995.

[bib28] Mollica RF, Caspi-Yavin Y, Bollini P, Truong T, Tor S, Lavelle J. The Harvard Trauma Questionnaire. Validating a cross-cultural instrument for measuring torture, trauma, and posttraumatic stress disorder in Indochinese refugees. J Nerv Ment Dis 1992; 180: 111–116. 1737972

[bib29] Wittchen HU, Schramm E, Zaudig M, Unland H. SKID Stukturiertes klinisches Interview für DSM-IV, Achse I, Deutsche Version (Structured Clinical Interview for DSM-IV, Axis I, German Version). Hogrefe: Göttingen, 1997..

[bib30] Wennig R. Potential problems with the interpretation of hair analysis results. Forensic Sci Int 2000; 107: 5–12. 10689559 10.1016/s0379-0738(99)00146-2

[bib31] Heeren M, Mueller J, Ehlert U, Schnyder U, Copiery N, Maier T. Mental health of asylum seekers: a cross-sectional study of psychiatric disorders. BMC Psychiatry 2012; 12: 114. 22900706 10.1186/1471-244X-12-114 PMC3490796

[bib32] Heeren M, Wittmann L, Ehlert U, Schnyder U, Maier T, Muller J. Psychopathology and resident status - comparing asylum seekers, refugees, illegal migrants, labor migrants, and residents. Compr Psychiatry 2014; 55: 818–825. 24636190 10.1016/j.comppsych.2014.02.003

[bib33] Dettenborn L, Tietze A, Kirschbaum C, Stalder T. The assessment of cortisol in human hair: associations with sociodemographic variables and potential confounders. Stress 2012; 15: 578–588. 22356099 10.3109/10253890.2012.654479

[bib34] Mewes R, Asbrock F, Laskawi J. Perceived discrimination and impaired mental health in Turkish immigrants and their descendents in Germany. Compr Psychiatry 2015; 62: 42–50. 26343466 10.1016/j.comppsych.2015.06.009

[bib35] Kalmakis KA, Meyer JS, Chiodo L, Leung K. Adverse childhood experiences and chronic hypothalamic-pituitary-adrenal activity. Stress 2015; 18: 446–450. 25783196 10.3109/10253890.2015.1023791

[bib36] Hinkelmann K, Muhtz C, Dettenborn L, Agorastos A, Wingenfeld K, Spitzer C et al. Association between childhood trauma and low hair cortisol in depressed patients and healthy control subjects. Biol Psychiatry 2013; 74: e15–e17. 23726317 10.1016/j.biopsych.2013.04.021

[bib37] Klengel T, Binder EB. Allele-specific epigenetic modification: a molecular mechanism for gene-environment interactions in stress-related psychiatric disorders? Epigenomics 2013; 5: 109–112. 23566086 10.2217/epi.13.11

[bib38] Ullmann E, Barthel A, Petrowski K, Stalder T, Kirschbaum C, Bornstein SR. Pilot study of adrenal steroid hormones in hair as an indicator of chronic mental and physical stress. Sci Rep 2016; 6: 25842. 27174654 10.1038/srep25842 PMC4865856

